# Gene alterations in the nuclear transport receptor superfamily: A study of head and neck cancer

**DOI:** 10.1371/journal.pone.0300446

**Published:** 2024-05-31

**Authors:** Phuong Thao Nguyen, Yudai Shimojukkoku, Yuka Kajiya, Yasunobu Oku, Ayami Tomishima, Kaori Shima, Tomonori Sasahira

**Affiliations:** 1 Department of Molecular Oral Pathology and Oncology, Graduate School of Medical and Dental Science, Kagoshima University, Kagoshima, Japan; 2 Department of Oral and Maxillofacial Surgery, Graduate School of Medical and Dental Science, Kagoshima University, Kagoshima, Japan; Tongji Medical University: Tongji Medical College, CHINA

## Abstract

In cancer cells, the nuclear transport system is often disrupted, leading to abnormal localization of nuclear proteins and altered gene expression. This disruption can arise from various mechanisms such as mutations in genes that regulate nuclear transport, altered expression of transport proteins, and changes in nuclear envelope structure. Oncogenic protein build-up in the nucleus due to the disturbance in nuclear transport can also boost tumor growth and cell proliferation. In this study, we performed bioinformatic analyses of 23 key nuclear transport receptors using genomic and transcriptomic data from pancancer and head and neck squamous cell carcinoma (HNSCC) datasets from The Cancer Genome Atlas (TCGA) and Cancer Cell Line Encyclopedia and found that the total alteration frequency of 23 nuclear transport receptors in 2691 samples of the PCAWG Consortium was 42.1% and a high levels of genetic alterations was significantly associated with poor overall survival. Amplification was the most common type of genetic alterations, and results in the overexpression of nuclear transport receptors in HNSCC compared to normal tissues. Furthermore, our study revealed that seven out of eight cell cycle genes (*CDK1*, *CDK2*, *CDK4*, *CDK6*, *CCNA1*, *CCNB1*, and *CCNE2*) were significantly and positively correlated with nuclear transport receptor genes in TCGA pancancer and CCLE datasets. Additionally, functional enrichment analysis showed that nuclear transport receptor genes were mainly enriched in the adhesion junction, cell cycle, ERBB, MAPK, MTOR and WNT signaling pathways.

## Introduction

Genetic mutations and chromosomal abnormalities are hallmarks of human cancers, including head and neck squamous cell carcinoma (HNSCC); HNSCC ranks as the sixth most prevalent cancer worldwide, with an annual incidence of approximately 930,000 cases and a mortality rate of approximately 50% [1]. While conventional treatments, such as radiation therapy, chemotherapy, surgery, novel immunotherapies, and combination therapies, are available, recurrence occurs in 50% of patients. In addition, tumor resection surgery can diminish patients’ physical function after surgery, but many patients still experience recurrence and metastasis [1,2]. Thus, the 5-year overall survival rate of HNSCCs remains unsatisfactory.

Nuclear transport receptors including importins and exportins are proteins that transport molecules from the cytoplasm to the nucleus and vice versa, which is crucial for many cellular processes, including gene expression, DNA replication, and repair [3]. In humans, there are at least 23 nuclear transport receptor family members [4,5], while the yeast *Saccharomyces cerevisiae* has 14 members. Nuclear transport receptors facilitate either the import or the export of molecules [3]. When proteins are imported into the nucleus, they interact with importin β directly or via an adapter molecule called importin α [6,7]. Importin α binds the nuclear localization signal (NLS) and forms a trimeric complex with importin β, which then targets the nuclear pore complex (NPC). The complex undergoes a series of interactions with the NPC before translocating into the nucleus [8,9]. Upon reaching the nucleus, the cargo is released to carry out its function, while the other transport factors are recycled for future transport cycles.

Cancer development, progression, and resistance to treatment have been linked to malfunctions in the nuclear-cytoplasmic transport system [10]. Specifically, karyopherin nuclear transport receptors, a crucial component of this system, are responsible for stabilizing chromosomes and supporting mitosis [10]. As a result, these receptors can impact the location of tumor suppressors and proto-oncogenes, thereby influencing the tumorigenesis process and drug sensitivity of cancer cells [10]. Given their importance in the development of cancer, there is a need for comprehensive molecular analysis of nuclear transport, which is currently lacking in the literature.

## Materials and methods

### Genetic alteration analysis

To investigate the genetic characteristics of nuclear transport receptor genes, we accessed the cBioPortal database (https://www.cbioportal.org/) [11,12], selected “TCGA Pan Cancer Atlas Studies” in the “Quick select” section and searched for gene names of 22 main nuclear transport receptor genes (S1 Table in S1 File) in (i) 2691 samples of PCAWG Consortium [13], (ii) 850 tumor cell lines of Cancer Cell Line Encyclopedia (CCLE) [14], (iii) 523 samples of TCGA-HNSCC (Pancancer atlas) [11,12], and (iv) 56 HNSCC and 78 non-cancerous cell lines of CCLE [14]. Within the “Cancer Types Summary” module, we reviewed the mutation type, alteration frequency, and CNA data across datasets. The tab OncoPrint showed an overview of the genetic alterations present in the 23 main nuclear transport receptor genes. Furthermore, the “Mutation” module provided a schematic diagram of the protein structure and detailed information on mutated sites.

### Analysis of gene alteration on patient survival

The correlation between gene alterations and patient survival was analyzed using the cBioPortal database [11,12]. We analyzed survival data for all tumor samples with or without genetic alterations in the “Comparison/Survival” module, with a log-rank *P* value < 0.05 indicating statistical significance.

### Gene set enrichment analysis (GSEA)

GSEA is a computational method for assessing whether a set of previously defined genes differ statistically significantly and consistently between two biological states [15]. GSEA was performed by GSEA software (version 4.3.0) to further investigate the functional enrichment of the genome under high expression conditions of 22 nuclear transport receptors (defined as higher than the median of mRNA levels). False detection rate (FDR) < 25% and nominal p < 0.05 were defined as the cutoff criteria.

### Statistical analysis

Independent-samples t test and Mann-Whitney U test were used to assess normal and skewed variables, respectively. Categorical variables were analyzed using the chi-square test or Fisher’s exact test, as appropriate. GraphPad Prism 9.3.1 software was used as the tool to visualize the results. P < 0.05 was considered statistically significant.

## Results

### Genetic alterations in nuclear transport receptor genes are widespread across cancers

**Fig 1:**
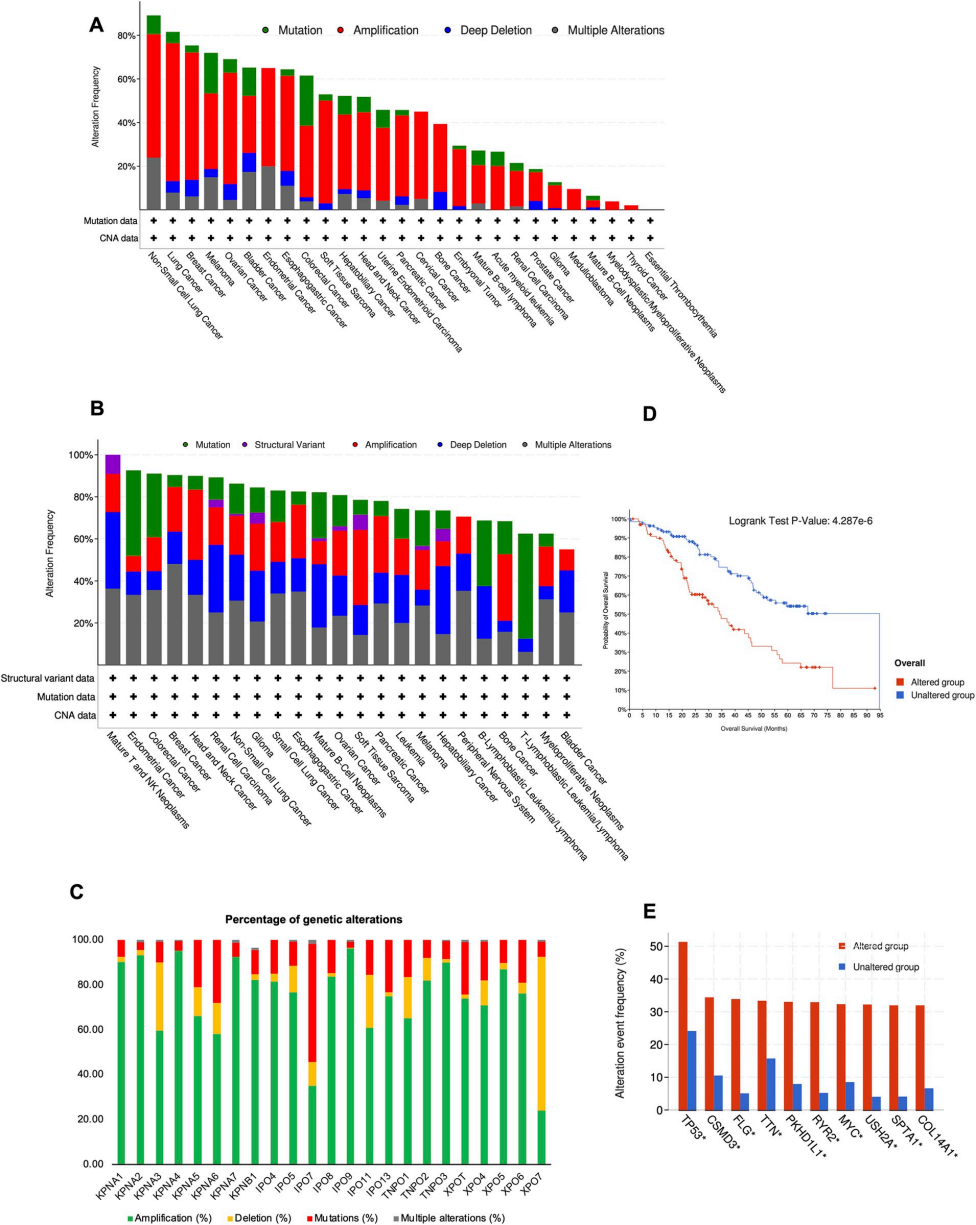
Genetic alterations of 23 nuclear transport receptors in pan-cancer. (A) in 2691 samples of PCAWG Consortium (B) in 850 tumor cell lines of CCLE dataset. (C) Genetic alterations of each nuclear transport receptor (*KPNA1*, *KPNA2*, *KPNA3*, *KPNA4*, *KPNA5*, *KPNA6*, *KPNA7*, *IPO5*, *IPO7*, *IPO8*, *IPO9*, *IPO11*, *IPO13*, *TNPO1*, *TNPO2*, *TNPO3*, *XPOT*, *XPO4*, *XPO5*, *XPO6*, and *XPO7*) in 2691 samples of PCAWG Consortium. (D) Comparation of overall survival rate between genetic altered group and unaltered group. (E) The significantly changed genes in genetic altered group and unaltered group.

Nuclear transport is dynamically mediated by nuclear transport receptors including importins and exportins. We initially used cBioPortal to evaluate genetic alterations in 23 main nuclear transport receptors (S1 Table in S1 File). This was conducted for 2691 samples from the PCAWG Consortium [13], and the tumour entities for these samples are listed in S2 Table in S1 File. We found that the total alteration frequency of this dataset was 42.1% (1133/2691). The frequencies of mutation, amplification, and deep deletion were 4.46% (120/2691), 30.17% (812/2691), and 3.27% (88/2691), respectively. Only 4.2% (113/2691) of these cases had two or more alterations (Fig 1A and S2 Table in S1 File).

In addition, we conducted similar genetic alteration analysis in 850 samples from CCLE [14], of which the tumour entities are listed in S3 Table in S1 File. The genetic alteration profiles of nuclear transport receptors for tumours in the CCLE database showed that alteration frequencies of mutation, structural variation, amplification and deep deletion were 15.76% (134/850), 1.41% (12/850), 19.88% (169/850), and 19.88% (169/850) respectively. A total of 29.65% (252/850) of these cell lines had two or more alterations (Fig 1B and S3 Table in S1 File). Among the types of genetic alterations, amplification was the most common type in nuclear transport receptors (58%-96.18%) (Fig 1C and S4 Table in S1 File), particularly in the karyopherin α family (*KPNA1*, *KPNA2*, *KPNA3*, *KPNA4*, *KPNA5*, *KPNA6*, and *KPNA7*). Among 23 main nuclear transport receptors, deletion was common in XPO7 (68.35%), while mutation was common in IPO7 (52.63%).

### Impact of genetic alterations of nuclear transport receptors on overall survival (OS)

Next, we analyzed the impact of genetic alterations in nuclear transport receptors on the survival rates of patients. The patients in the PCAWG pancancer dataset were categorized into two groups: the alteration group and no alteration group. To compare the survival rates between 2 groups, the log-rank (Mantel-Cox) test was applied, and Kaplan-Meier survival curves were generated. Interestingly, a high frequency of genetic alterations in 23 main nuclear transport receptors was significantly associated with poor OS (Fig 1D). Furthermore, *TP53*, *CSMD3*, *FLG*, *TTN*, *PKHD1L1*, *RYR2*, *MYC*, *USH2A*, *SPTA1*, and *COL14A1* were significantly upregulated in the group with genetic alterations in the nuclear transport receptors compared to the group without alterations in nuclear transport receptors compared to the group without alterations (Fig 1E).

**Fig 2:**
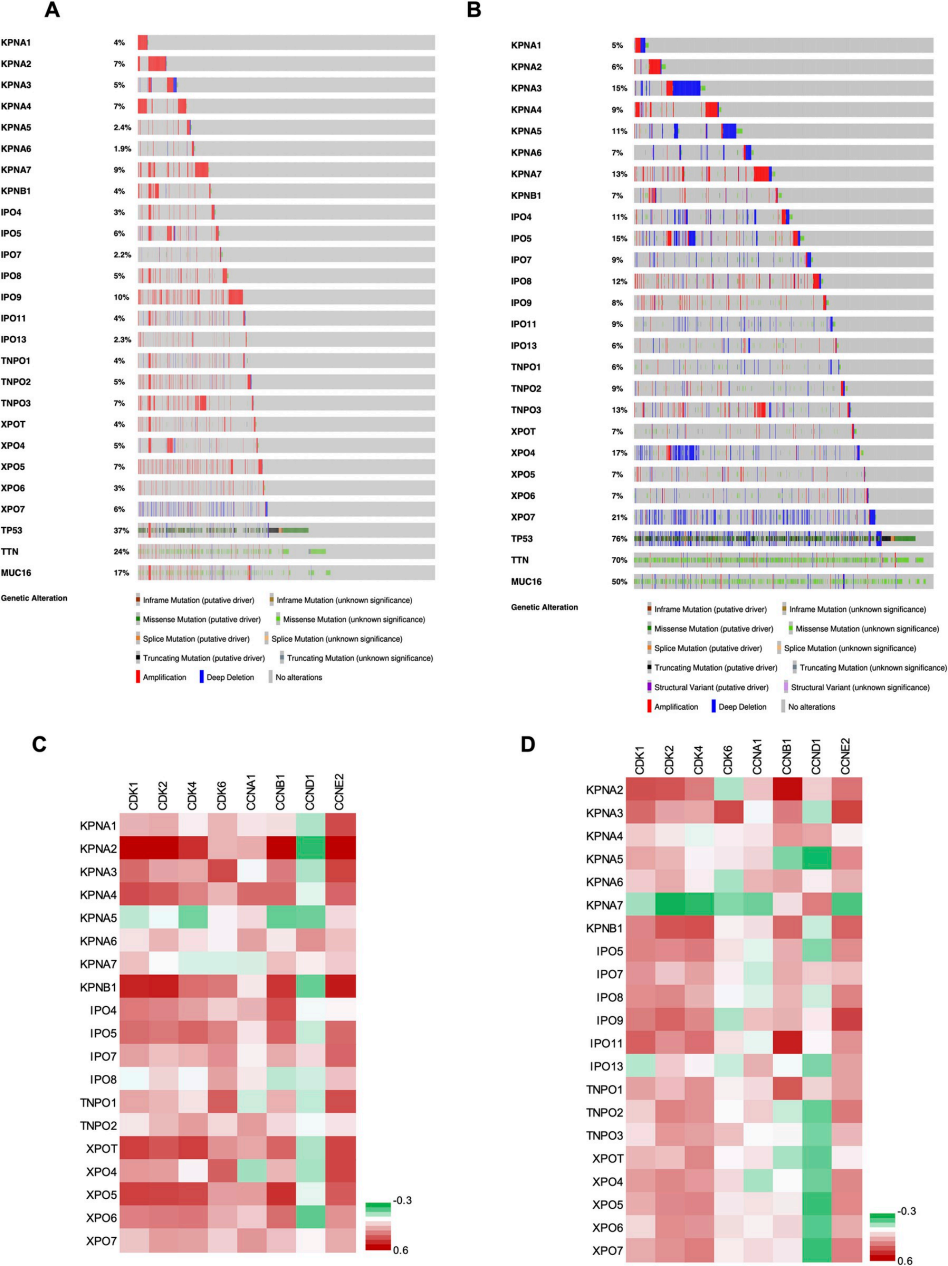
Genetic alteration of 23 selected nuclear transport receptors in (A) 27 tumour types from the Pan-Cancer Analysis of Whole Genomes (PCAWG) Consortium of the International Cancer Genome Consortium (ICGC) and The Cancer Genome Atlas (TCGA) and (B) 850 cell lines of Cancer Cell Line Encyclopedia. Cell cycle regulation genes predominantly show a positive correlation with nuclear transport receptors. Heatmap showing positive (co-expression) or negative (mutual exclusivity) correlation between cell cycle genes and nuclear transport receptor genes in (C) the TCGA pan-cancer cohort and (D) CCLE. Spearman’s rank correlation coefficient is shown as colour scale at right side.

Among 23 main nuclear transport receptor genes, the genetic alteration percentage in the PCAWG Consortium ranged from 1.9% (in *KPNA6*) to 10% (in *IPO9*) (Fig 2A), and in the CCLE dataset the genetic alteration percentage ranged from 5% (in *KPNA1*) to 21% (in *XPO7*) (Fig 2B). We interestingly found that cases with alterations in nuclear transport receptor genes also had mutations in top driver genes like *TP53*, *TTN*, and *MUC16* (Fig 2A and 2B). Furthermore, analysis of mutations in the transport receptors and top driver genes showed that some mutations tended to co-occur, while others appeared to be mutually exclusive (Table 1). Somatic mutations including missense mutations, truncating mutations (nonsense, nonstop, frameshift deletion, frameshift insertion, splice site), in-frame mutations (in-frame deletion, in-frame insertion) and all other mutations in all the transporters, are shown in S1 Fig in S1 File and S5 Table in S1 File.

**Table 1:** Co-occurring and mutually exclusion mutations in nuclear transport receptors and top mutated genes in PCAWG pancancer dataset.

A	B	Neither	A Not B	B Not A	Both	Log2 Odds Ratio	p-Value	q-Value	Tendency
KPNA3	XPO4	696	61	81	75	>3	<0.001	<0.001	Co-occurrence
KPNA7	TNPO3	736	59	61	57	>3	<0.001	<0.001	Co-occurrence
KPNA3	IPO5	701	71	76	65	>3	<0.001	<0.001	Co-occurrence
IPO5	XPO4	673	84	99	57	2.206	<0.001	<0.001	Co-occurrence
KPNA1	KPNA4	805	25	61	22	>3	<0.001	<0.001	Co-occurrence
IPO11	TNPO1	806	57	28	22	>3	<0.001	<0.001	Co-occurrence
KPNA2	KPNB1	805	37	51	20	>3	<0.001	<0.001	Co-occurrence
TNPO2	MUC16	428	13	405	67	2.445	<0.001	<0.001	Co-occurrence
KPNA6	IPO13	811	46	38	18	>3	<0.001	<0.001	Co-occurrence
TTN	MUC16	169	272	107	365	1.084	<0.001	<0.001	Co-occurrence
IPO8	MUC16	410	31	389	83	1.497	<0.001	<0.001	Co-occurrence
KPNA5	XPO5	767	81	45	20	2.073	<0.001	<0.001	Co-occurrence
IPO9	XPO7	680	43	158	32	1.679	<0.001	<0.001	Co-occurrence
XPO7	MUC16	375	66	348	124	1.018	<0.001	<0.001	Co-occurrence
KPNB1	XPO7	681	42	161	29	1.546	<0.001	0.001	Co-occurrence
KPNB1	IPO9	783	55	59	16	1.949	<0.001	0.001	Co-occurrence
IPO4	XPO7	654	69	151	39	1.292	<0.001	0.001	Co-occurrence
KPNA2	IPO9	795	43	61	14	2.085	<0.001	0.002	Co-occurrence
IPO9	XPO6	784	60	54	15	1.86	<0.001	0.004	Co-occurrence
TNPO1	TNPO3	761	34	102	16	1.812	<0.001	0.004	Co-occurrence
KPNA3	IPO4	699	106	78	30	1.343	<0.001	0.004	Co-occurrence
IPO9	IPO11	775	59	63	16	1.738	<0.001	0.004	Co-occurrence
IPO9	XPO5	787	61	51	14	1.824	<0.001	0.006	Co-occurrence
TNPO1	XPO5	809	39	54	11	2.079	<0.001	0.006	Co-occurrence
KPNB1	XPOT	792	58	50	13	1.828	<0.001	0.008	Co-occurrence
IPO11	TNPO3	737	58	97	21	1.46	<0.001	0.008	Co-occurrence
IPO11	TNPO2	770	63	64	16	1.611	<0.001	0.008	Co-occurrence
KPNA4	TP53	214	8	616	75	1.703	<0.001	0.008	Co-occurrence
KPNA6	KPNB1	791	51	58	13	1.798	<0.001	0.008	Co-occurrence
TNPO2	XPO5	782	66	51	14	1.702	<0.001	0.008	Co-occurrence
KPNB1	XPO5	790	58	52	13	1.768	<0.001	0.008	Co-occurrence
KPNA6	IPO4	758	47	91	17	1.591	<0.001	0.008	Co-occurrence
IPO11	MUC16	417	24	417	55	1.196	<0.001	0.009	Co-occurrence
IPO4	XPO5	757	91	48	17	1.559	<0.001	0.009	Co-occurrence
IPO9	XPOT	788	62	50	13	1.724	0.001	0.009	Co-occurrence
KPNA2	XPOT	804	46	52	11	1.886	0.001	0.009	Co-occurrence
IPO7	TNPO2	768	65	64	16	1.563	0.001	0.012	Co-occurrence
IPO7	XPO7	671	52	161	29	1.217	0.001	0.012	Co-occurrence
IPO7	XPO6	777	67	55	14	1.562	0.002	0.014	Co-occurrence
IPO11	XPO5	782	66	52	13	1.567	0.002	0.019	Co-occurrence
KPNB1	IPO13	797	60	45	11	1.699	0.003	0.02	Co-occurrence
KPNA5	IPO4	726	79	86	22	1.233	0.003	0.021	Co-occurrence
KPNB1	TNPO2	776	57	66	14	1.53	0.003	0.024	Co-occurrence
KPNA4	TNPO1	791	72	39	11	1.632	0.004	0.026	Co-occurrence
TNPO1	XPO7	692	31	171	19	1.311	0.004	0.027	Co-occurrence
KPNA2	XPO7	687	36	169	21	1.246	0.004	0.027	Co-occurrence
KPNA1	XPO4	726	31	140	16	1.42	0.004	0.029	Co-occurrence
IPO9	TNPO2	772	61	66	14	1.425	0.004	0.029	Co-occurrence
KPNB1	TTN	265	11	577	60	1.325	0.004	0.029	Co-occurrence
IPO11	TTN	263	13	571	66	1.226	0.005	0.03	Co-occurrence
IPO4	TNPO2	743	90	62	18	1.261	0.006	0.035	Co-occurrence
IPO8	IPO11	738	96	61	18	1.182	0.007	0.043	Co-occurrence
KPNB1	XPO6	785	59	57	12	1.486	0.008	0.047	Co-occurrence
IPO5	IPO8	686	113	86	28	0.983	0.008	0.047	Co-occurrence
KPNA7	IPO4	712	93	85	23	1.051	0.008	0.048	Co-occurrence
XPOT	XPO4	713	44	137	19	1.168	0.008	0.048	Co-occurrence

### Genetic alteration of nuclear transport receptor genes is significantly associated with cell cycle activation

Cell cycle activation is a crucial aspect of cancer development, and its regulation is governed by various genes [16]. Thus, we evaluated the relationship between the expression of nuclear transport receptors and cell cycle regulation genes (*CDK1*, *CDK2*, *CDK4*, *CDK6*, *CCNA1*, *CCNB1*, *CCND1*, and *CCNE2*) using cBioPortal. Most nuclear transport receptor genes showed a significant positive correlation with seven of eight cell cycle genes (*CDK1*, *CDK2*, *CDK4*, *CDK6*, *CCNA1*, *CCNB1*, and *CCNE2*) in the TCGA pancancer and CCLE datasets (Fig 2C and 2D) with *q*-values shown in S6 and S7 Tables in S1 File.

### Genetic alterations in nuclear transport receptor genes are widespread in HNSCC

**Fig 3:**
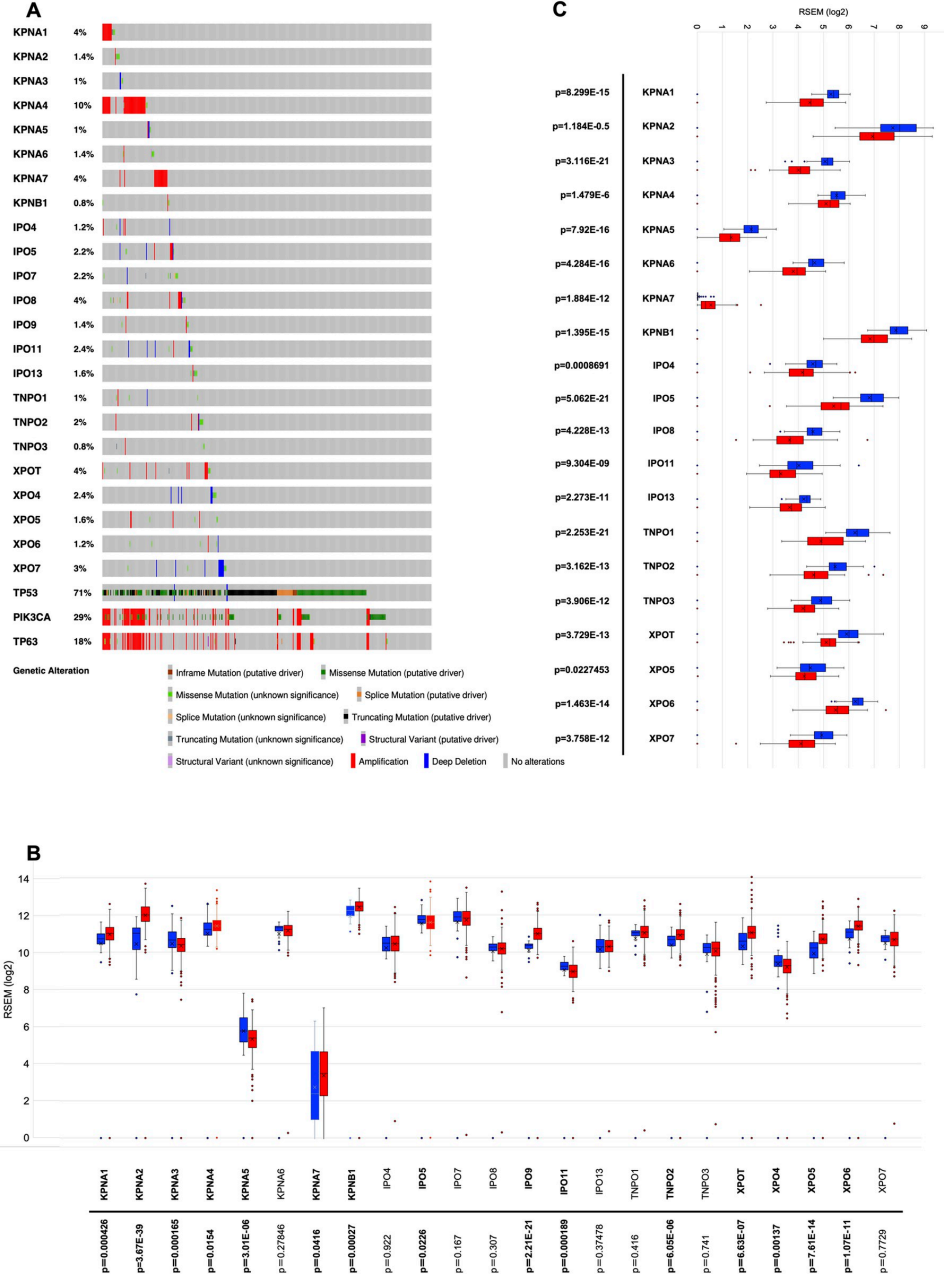
Genetic alteration of 23 nuclear transport receptors in HNSCC patient samples of TCGA dataset (A). Their expression in tumor tissue vs. normal tissues (B) and in HNSCC cell lines of CCLE dataset (C).

Furthermore, we again performed Oncoprint analysis through cBioportal’s OncoPrint tool to assess 23 nuclear receptor genes in 523 primary head and neck tumour samples, and then compared the mutational landscape of transport-related genes in HNSCC. The genetic alteration percentage in HNSCC ranged from 0.8% (in *TNPO3*) to 10% (in *KPNA4*) (Fig 3A). Somatic mutations in 23 transporters in HNSCC are shown in S8 Table in S1 File and Fig 3A. Interestingly, we found that the cases in which nuclear transport receptor genes were altered also expressed the top mutated driver genes, including *TP53*, *PIK3CA* and *TP63* (Fig 3A). Moreover, among the alterations in several transport receptors and these top mutated genes, some mutations were co-occurring and some were mutual exclusive (Table 2).

**Table 2:** Co-occurring and mutually exclusion mutations in nuclear transport receptors and top mutated genes in HNSCC.

A	B	Neither	A not B	B not A	Both	Log2 Odds Ratio	p-Value	q-Value	Tendency
KPNA4	TP63	398	11	48	39	>3	<0.001	<0.001	Co-occurrence
KPNA4	PIK3CA	347	4	99	46	>3	<0.001	<0.001	Co-occurrence
PIK3CA	TP63	329	80	22	65	>3	<0.001	<0.001	Co-occurrence
KPNA1	KPNA4	439	7	38	12	>3	<0.001	<0.001	Co-occurrence
KPNA1	TP63	403	6	74	13	>3	<0.001	<0.001	Co-occurrence
KPNA1	PIK3CA	345	6	132	13	2.502	<0.001	0.019	Co-occurrence
KPNA7	TP53	143	0	331	22	>3	<0.001	0.021	Co-occurrence
IPO4	TNPO3	488	4	2	2	>3	<0.001	0.029	Co-occurrence
KPNA2	TNPO1	486	5	3	2	>3	0.002	0.061	Co-occurrence
KPNB1	XPOT	476	2	16	2	>3	0.007	0.233	Co-occurrence
KPNA4	IPO4	443	47	3	3	>3	0.016	0.459	Co-occurrence
XPOT	XPO6	474	16	4	2	>3	0.017	0.464	Co-occurrence
IPO4	IPO8	473	4	17	2	>3	0.019	0.476	Co-occurrence
TP53	TP63	126	283	17	70	0.874	0.022	0.504	Co-occurrence
KPNA1	IPO8	461	16	16	3	2.434	0.031	0.679	Co-occurrence
XPO5	TP63	405	4	83	4	2.287	0.035	0.715	Co-occurrence
TNPO1	TNPO3	488	4	3	1	>3	0.04	0.762	Co-occurrence

### Gene expression analysis

As shown in the above results, amplification was the most common genetic alteration in nuclear transport receptor genes. It is well known that gene amplification is a common feature in many human cancers, and overexpression of genes due to amplification is a frequent occurrence in cancer [17]. Thus, we examined the expression patterns of nuclear transport receptors in normal tissues and the tumors of the TCGA-HNSCC dataset, and the results are shown in Fig 3B. The expression of *KPNA1*, *KPNA2*, *KPNA4*, *KPNA7*, *KPNB1*, *IPO9*, *TNPO2*, *XPOT*, *XPO5* and *XPO6* was significantly higher in tumour tissue than in normal tissues, whereas the expression of *KPNA3*, *KPNA5*, *IPO5*, *IPO11* and *XPO4* was significantly lower in tumor tissues than in the normal tissues (Fig 3B). Next, we analyzed the relative expression of nuclear transport receptor genes in 56 HNSCC and 78 non-cancerous cell lines from CCLE. Our analysis revealed KPNA7 as a nuclear transport receptor overexpressed in HNSCC tumors relative to normal tissues, based on the TCGA-HNSCC dataset. Likewise, we also found KPNA7 to exhibit higher expression in HNSCC cell lines compared to non-cancerous cell lines (Fig 3C).

### Functional enrichment analysis

**Fig 4:**
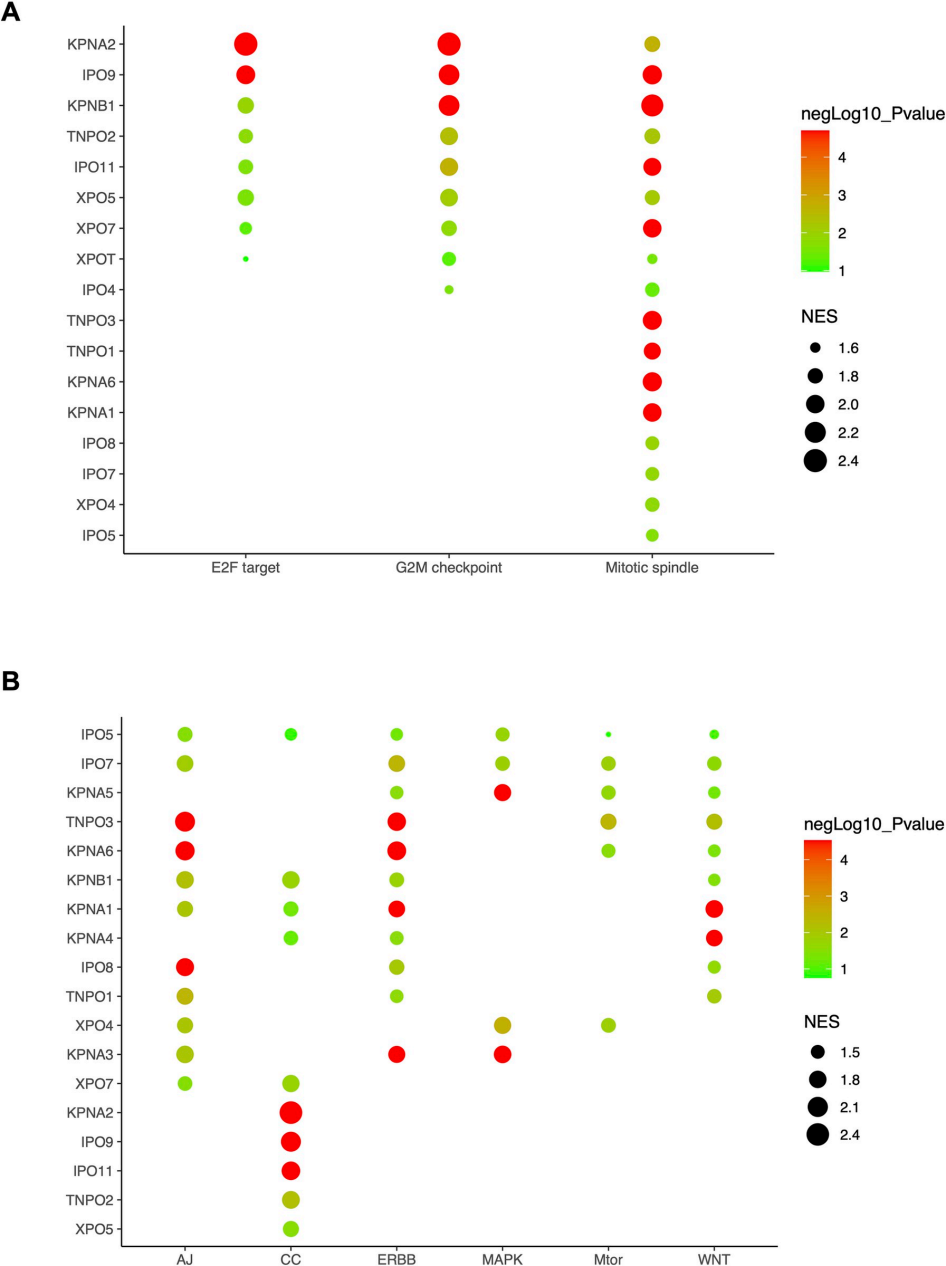
Functional enrichment analysis of 23 nuclear transport receptors in HNSCC (A) Enrichment analyses of Hallmarks (B) Enrichment analyses of KEGG.

To gain insight into the known biological processes involved in HNSCC, cancer hallmark and KEGG pathway enrichment analysis (GSEA) were performed on expression data of selected nuclear transport receptor genes. According to the hallmark results, these genes were mainly enriched in E2F targets, G2M checkpoint and mitotic spindle (Fig 4A).

According to the results of KEGG signaling pathway analysis, nuclear transport receptor genes were mainly enriched in the adhesion junction (AJ), cell cycle (CC), ERBB, MAPK, MTOR and WNT signaling pathways (Fig 4B).

### Impact of genetic alterations of nuclear transport receptors on overall survival (OS)

**Fig 5:**
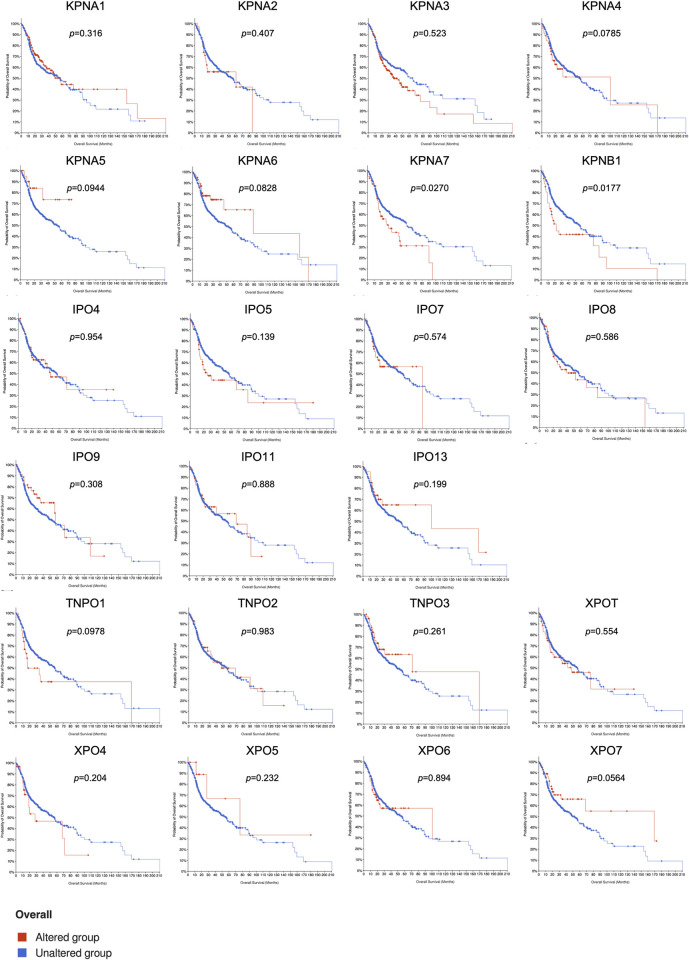
Meier-Kaplan plots showing the correlation between the expression of 23 nuclear transport receptors and overall HNSCC patient survival.

Next, we analyzed the impact of genetic alterations of each nuclear transport receptor on the survival rates of HNSCC patients. Interestingly, we found that alterations in some of the members, such as KPNA7 and KPNB1, showed a significant correlation with the patient survival (Fig 5).

## Discussion

Eukaryotic cells are characterized by a nuclear membrane that separates the nuclear and cytoplasmic components, which require a set of specialized transporters that transport molecules to and from the nucleus to the cytoplasm to ensure cellular homeostasis. Nuclear transport receptors play a crucial role in regulating the cell cycle by interacting with chromatin and genes associated with cell cycle progression; for example, p53, a protein crucial to the stress response, must be localized in the nuclear to function, and its nuclear localization is tightly regulated by both nuclear import and nuclear export of p53 [18,19]. Although p53 is synthesized in the cytoplasm, it regulates transcription in the nucleus. However, the precise signals or proteins that direct p53’s movement from the cytoplasm to the nucleus remain unclear. Many tumor types exhibit the abnormal cytoplasmic sequestration of p53 and display poor responses to chemotherapy and radiation therapies, which has led researchers to explore which of the major skeletal filament systems (such as actin filaments, intermediate filaments, or microtubules) could serve as a cytoplasmic anchor for p53 molecules [20–22]. p53 molecules are imported into the nucleus via their three nuclear localization signals (NLS) [23,24] and exported via their two nuclear export signals (NES) [25,26]. Following DNA damage, p53 is imported into the nucleus through its NLS [27]. Recently, importin α3 was discovered to regulate the nucleocytoplasmic shuttling and activity of p53 [28].

EGFR, a renowned receptor tyrosine kinase, can be translocated into different organelles, including the nucleus and mitochondrion, upon stimuli such as ligand binding, radiation, and EGFR-targeted therapy [29]. Nuclear EGFR is a multifunctional regulator with roles as a transcriptional regulator, tyrosine kinase, and mediator of other physiological processes [29]. Studies have shown that nuclear EGFR is an indicator of poor clinical outcomes in cancer patients [30,31]. Moreover, nuclear EGFR has been shown to contribute to resistance to various cancer therapies, such as radiation, cisplatin, and cetuximab [32–34].

In a previous study, we observed that a significant increase in FGFR1 nuclear localization in HNSCC corresponded with high-grade histopathology, abundant nuclear polymorphisms and a high-grade invasion pattern [35]. Stachowiak et al revealed that nuclear FGFR1 facilitates the transition from G0/G1 to S phase of the cell cycle [36]. Moreover, nuclear FGFR1 initiates the release of CREB-binding protein (CBP) from its inactive complex with RSK1 [37], thereby increasing gene activities linked to cellular differentiation. Additionally, the protease granzymeB (GrB) is responsible for the cleavage of FGFR1, leading to the nuclear localization of FGFR1 cleavage and the invasion of breast cancer cells into the stroma [38].

Various studies have highlighted that nuclear transport receptors including XPO1, KPNA2, and KPNA4, exhibit hyperactivity in cancer and facilitate the export of vital tumor suppressors to the cytoplasm [39] or the import of oncogenes to the nucleus [40]. Although there have been no reports on the impact of their mutations and amplifications and mutations on outcomes in NSCLC, XPO1 has been shown to be involved in the development of other cancer types [5]. Researchers have been exploring several approaches to target nuclear transport, including the use of small molecule inhibitors such as KPT-330 and KPT-8602, which have shown promise in preclinical models of various cancers [41,42]. Additionally, inhibiting nuclear pore complex proteins such as Nup98 and Nup214 has emerged as a potential therapeutic approach [43]. Gene therapy approaches such as siRNA-mediated knockdown of XPO1 and CRISPR-Cas9 technology have also shown promise in preclinical models of head and neck cancer [43–45]. Finally, the nuclear transport of the immune checkpoint molecule PD-L1 has been linked to the regulation of T-cell activity in the tumor microenvironment [46].

In conclusion, the significance of nuclear transport in cancer biology cannot be overstated. It is involved in key processes such as gene expression, DNA repair, cell cycle regulation, and immunotherapy. Dysregulation of nuclear transport is a defining characteristic of cancer and can lead to the advancement of tumors. Focusing on nuclear transport as a therapeutic target can lead to the development of innovative cancer treatments, further improving patient outcomes.

## Supporting information

S1 File (DOCX)
