# Supplementary material for: Gene alterations in the nuclear transport receptor superfamily: A study of head and neck cancer
Source: PLoS One. 2024 May 31;19(5):e0300446. doi: 10.1371/journal.pone.0300446 (PMC11142601; doi:10.1371/journal.pone.0300446)
Supplement: S1 File — (DOCX) [file pone.0300446.s001.docx]

**SUPPORTING INFORMATION**

Supplemental Figure 1: Scheme of genetic alterations in nuclear transport receptors in pan-cancer

Supplemental Table 1: List of 23 main nuclear transport receptors

| **Nuclear transport receptor** | **Gene name** |
| --- | --- |
| Karyopherin α1 | *KPNA1* |
| Karyopherin α2 | *KPNA2* |
| Karyopherin α3 | *KPNA3* |
| Karyopherin α4 | *KPNA4* |
| Karyopherin α5 | *KPNA5* |
| Karyopherin α6 | *KPNA6* |
| Karyopherin α7 | *KPNA7* |
| Karyopherin β1 | *KPNB1* |
| Importin 4 | *IPO4* |
| Importin 5 | *IPO5* |
| Importin 7 | *IPO7* |
| Importin 8 | *IPO8* |
| Importin 9 | *IPO9* |
| Importin 11 | *IPO11* |
| Importin 13 | *IPO13* |
| Transportin 1 | *TNPO1* |
| Transportin 2 | *TNPO2* |
| Transportin 3 | *TNPO3* |
| Exportin T | *XPOT* |
| Exportin 4 | *XPO4* |
| Exportin 5 | *XPO5* |
| Exportin 6 | *XPO6* |
| Exportin 7 | *XPO7* |

Supplemental Table 2: Genetic alterations of 23 nuclear transport receptors in various cancer types of PCAWG Consortium

| **Cancer types** | **Mutation** | **Amplification** | **Deep Deletion** | **Multiple Alterations** | **Total** |
| --- | --- | --- | --- | --- | --- |
| Non-Small Cell Lung Cancer (n=46) | 3 (6.52%) | 26 (56.52%) | 0 | 11 (23.91%) | 40 (86.96%) |
| Lung Cancer (n=38) | 2 (5.26%) | 24 (63.16%) | 2 (5.26%) | 3 (7.89%) | 31 (81.58%) |
| Breast Cancer (n=211) | 6 (2.84%) | 117 (55.45%) | 16 (7.58%) | 13 (6.16%) | 152 (72.04%) |
| Melanoma (n=107) | 21 (19.63%) | 37 (34.58%) | 4 (3.74%) | 15 (14.02%) | 77 (71.96%) |
| Ovarian Cancer (n=110) | 6 (5.45%) | 56 (50.91%) | 8 (7.27%) | 5 (4.55) | 75 (68.18%) |
| Bladder Cancer (n=23) | 3 (13.04%) | 6 (26.09%) | 2 (8.7%) | 4 (17.39%) | 15 (65.22%) |
| Esophagogastric Cancer (n=163) | 5 (3.07%) | 73 (44.79%) | 11 (6.75%) | 16 (9.82%) | 105 (64.42%) |
| Colorectal Cancer (n=52) | 12 (23.08%) | 17 (32.69%) | 1 (1.92%) | 2 (3.85%) | 32 (61.54%) |
| Endometrial Cancer (n=12) | 0 | 8 (40%) | 0 | 4 (20%) | 12 (60%) |
| Soft Tissue Sarcoma (n=34) | 1 (2.94%) | 16 (47.06%) | 1 (2.94%) | 0 | 18 (52.94%) |
| Hepatobiliary Cancer (n=358) | 29 (8.1%) | 123 (34.36%) | 8 (2.23%) | 23 (6.42%) | 183 (51.12%) |
| Head and Neck Cancer (n=56) | 3 (5.36%) | 20 (35.71%) | 2 (3.57%) | 3 (5.36%) | 28 (50%) |
| Uterine Endometrioid Carcinoma (n=103) | 5 (4.85%) | 17 (16.5%) | 0 | 2 (1.94%) | 24 (45.83%) |
| Cervical Cancer (n=20) | 0 | 8 (40%) | 0 | 1 (5%) | 9 (45%) |
| Pancreatic Cancer (n=308) | 7 (2.27%) | 109 (35.39%) | 14 (4.55%) | 6 (1.95%) | 136 (44.16%) |
| Bone Cancer (n=24) | 0 | 19 (31.15%) | 5 (5.82%) | 0 | 24 (39.34%) |
| Embryonal Tumour (n=119) | 1 (0.84%) | 31 (26.05%) | 2 (1.68%) | 0 | 34 (28.57%) |
| Acute myeloid leukemia (n=15) | 1 (6.67%) | 3 (20%) | 0 | 0 | 4 (26.67%) |
| Mature B-cell lymphoma (n=103) | 5 (4.85%) | 17 (16.5%) | 0 | 2 (1.94%) | 24 (23.3%) |
| Renal Cell Carcinoma (n=168) | 6 (3.23%) | 28 (15.05%) | 0 | 3 (1.61%) | 37 (19.89%) |
| Prostate Cancer (n=273) | 2 (0.73%) | 35 (12.82%) | 10 (3.66%) | 0 | 47 (17.22%) |
| Glioma (n=134) | 1 (0.75%) | 14 (10.45%) | 1 (0.75%) | 0 | 16 (11.94%) |
| Medulloblastoma (n=21) | 0 | 2 (9.52%) | 0 | 0 | 2 (9.52%) |
| Mature B-Cell Neoplasms (n=93) | 1 (1.08%) | 3 (3.23%) | 1 (1.08%) | 0 | 5 (5.38%) |
| Myelodysplastic Neoplasms (n=26) | 0 | 1 (3.85%) | 0 | 0 | 1 (3.85%) |
| Thyroid Cancer (n=48) | 0 | 2 (2.08%) | 0 | 0 | 2 (2.08%) |
| Essential Thrombocythemia (n=26) | 0 | 0 | 0 | 0 | 0 |
| **Total** | 120 (4.46%) | 812 (30.17%) | 88 (3.27%) | 113 (4.2%) | 1133 (42.1%) |

Supplemental Table 3: Genetic alterations of 23 nuclear transport receptors in various cancer types of CCLE dataset

| **Cancer types** | **Mutation** | **Structural Variant** | **Amplification** | **Deep deletion** | **Multiple Alterations** | **Total** |
| --- | --- | --- | --- | --- | --- | --- |
| Mature T and NK Neoplasms (n=11) | 0 | 1 (9.09%) | 2 (18.18%) | 4 (36.36%) | 4 (36.36%) | 11 (100%) |
| Endometrial Cancer (n=27) | 11 (40.74%) | 0 | 2 (7.41%) | 3 (11.11%) | 9 (33.33%) | 25 (92.59%) |
| Colorectal Cancer (n=56) | 17 (30.36%) | 0 | 9 (16.07%) | 5 (8.93%) | 20 (35.71%) | 51 (91.07%) |
| Breast Cancer (n=52) | 3 (5.77%) | 0 | 11 (21.15%) | 8 (15.38%) | 25 (48.08%) | 47 (90.38%) |
| Head and Neck Cancer (n=30) | 2 (6.67%) | 0 | 10 (33.33%) | 5 (16.67%) | 10 (33.33%) | 27 (90%) |
| Renal Cell Carcinoma (n=28) | 3 (10.71%) | 1 (3.57%) | 5 (17.86%) | 9 (32.14%) | 7 (25%) | 25 (89.29%) |
| Non-Small Cell Lung Cancer (n=124) | 18 (14.52%) | 1 (0.81%) | 23 (18.55%) | 27 (21.77%) | 38 (30.65%) | 107 (86.29%) |
| Glioma (n=58) | 7 (12.07%) | 3 (5.17%) | 13 (22.41%) | 14 (24.14%) | 12 (20.69%) | 49 (84.48%) |
| Small Cell Lung Cancer (n=53) | 8 (15.09%) | 0 | 10 (18.87%) | 8 (15.09%) | 18 (33.96%) | 44 (83.02%) |
| Esophagogastric Cancer (n=63) | 4 (6.35%) | 0 | 16 (25.4%) | 10 (15.87%) | 22 (34.92%) | 52 (82.54%) |
| Mature B-Cell Neoplasms (n=73) | 16 (21.92%) | 1 (1.37%) | 8 (10.96%) | 22 (30.14%) | 13 (17.81%) | 60 (82.19%) |
| Ovarian Cancer (n=47) | 7 (14.89%) | 1 (2.13%) | 10 (21.28%) | 9 (19.15%) | 11 (23.4%) | 38 (80.85%) |
| Soft Tissue Sarcoma (n=14) | 1 (7.14%) | 1 (7.14%) | 5 (35.71%) | 2 (14.29%) | 2 (14.29%) | 11 (78.57%) |
| Pancreatic Cancer (n=41) | 3 (7.32%) | 0 | 11 (26.83%) | 6 (14.63%) | 12 (29.27%) | 32 (78.05%) |
| Leukemia (n=35) | 5 (14.29%) | 0 | 6 (17.14%) | 8 (22.86%) | 7 (20%) | 26 (74.29%) |
| Melanoma (n=53) | 9 (16.98%) | 1 (1.89%) | 10 (18.87%) | 4 (7.55%) | 15 (28.3%) | 39 (73.58%) |
| Hepatobiliary Cancer (n=34) | 3 (8.82%) | 2 (5.88%) | 4 (11.76%) | 11 (32.35%) | 5 (14.71%) | 25 (73.53%) |
| Peripheral Nervous System (n=17) | 0 | 0 | 3 (17.65%) | 3 (17.65%) | 6 (35.29%) | 12 (70.59%) |
| B-Lymphoblastic Leukemia/Lymphoma (n=16) | 5 (31.25%) | 0 | 0 | 4 (25%) | 2 (12.5%) | 11 (68.75%) |
| Bone Cancer (n=19) | 3 (15.79%) | 0 | 6 (31.58%) | 1 (5.26%) | 3 (15.79%) | 13 (68.42%) |
| T-Lymphoblastic Leukemia/Lymphoma (n=16) | 8 (50%) | 0 | 0 | 1 (6.25%) | 1 (6.25%) | 10 (62.5%) |
| Myeloproliferative Neoplasms (n=16) | 1 (6.25%) | 0 | 3 (18.75%) | 1 (6.25%) | 5 (31.25%) | 10 (62.5%) |
| Bladder Cancer (n=20) | 0 | 0 | 2 (10%) | 4 (20%) | 5 (25%) | 11 (55%) |
| **Total** | 134 (15.76%) | 12 (1.41%) | 169 (19.88%) | 169 (19.88%) | 252 (29.65%) | 736 (86.59%) |

Supplemental Table 4: Genetic alterations of each nuclear transport receptor

|  | **Amplification (%)** | **Deletion (%)** | **Mutations (%)** | **Multiple alterations (%)** |
| --- | --- | --- | --- | --- |
| KPNA1 | 90.32 | 2.15 | 7.53 |  |
| KPNA2 | 93.30 | 2.23 | 3.35 | 1.12 |
| KPNA3 | 59.54 | 30.53 | 9.16 | 0.76 |
| KPNA4 | 95.29 | 0.00 | 4.12 | 0.59 |
| KPNA5 | 66.13 | 12.90 | 20.97 | 0.00 |
| KPNA6 | 58.00 | 14.00 | 28.00 | 0.00 |
| KPNA7 | 92.38 | 0.00 | 6.28 | 1.35 |
| KPNB1 | 82.14 | 2.67 | 10.74 | 0.89 |
| IPO4 | 81.40 | 3.49 | 15.12 | 0.00 |
| IPO5 | 76.71 | 11.64 | 10.96 | 0.68 |
| IPO7 | 35.09 | 10.53 | 52.63 | 1.75 |
| IPO8 | 83.69 | 1.42 | 14.89 | 0.00 |
| IPO9 | 96.18 | 0.35 | 2.78 | 0.69 |
| IPO11 | 60.91 | 23.64 | 15.45 | 0.00 |
| IPO13 | 75.00 | 1.67 | 23.33 | 0.00 |
| TNPO1 | 65.05 | 18.45 | 16.50 | 0.00 |
| TNPO2 | 81.88 | 10.14 | 7.97 | 0.00 |
| TNPO3 | 90.00 | 1.58 | 7.89 | 0.53 |
| XPOT | 73.87 | 1.80 | 23.42 | 0.90 |
| XPO4 | 70.87 | 11.02 | 17.32 | 0.79 |
| XPO5 | 87.03 | 2.70 | 10.27 | 0.00 |
| XPO6 | 76.19 | 4.76 | 19.05 | 0 |
| XPO7 | 24.05 | 68.35 | 6.96 | 0.63 |

Supplemental Table 5: Mutation types of each nuclear transport receptor in PCAWG consortium

|  | Missense | Truncating | Inflame | Splice | Fusion | Total | % |
| --- | --- | --- | --- | --- | --- | --- | --- |
| KPNA1 | 7 | 1 | 0 | 0 | 0 | 8 | 0.3 |
| KPNA2 | 7 | 0 | 0 | 1 | 0 | 8 | 0.3 |
| KPNA3 | 9 | 2 | 0 | 2 | 0 | 13 | 0.5 |
| KPNA4 | 5 | 2 | 0 | 1 | 0 | 8 | 0.3 |
| KPNA5 | 13 | 1 | 0 | 0 | 0 | 14 | 0.5 |
| KPNA6 | 12 | 0 | 0 | 2 | 0 | 14 | 0.5 |
| KPNA7 | 14 | 1 | 1 | 2 | 0 | 18 | 0.6 |
| IPO4 | 13 | 0 | 0 | 0 | 0 | 13 | 0.5 |
| IPO5 | 13 | 3 | 1 | 0 | 0 | 17 | 0.6 |
| IPO7 | 24 | 6 | 0 | 3 | 0 | 33 | 1.3 |
| IPO8 | 16 | 8 | 0 | 1 | 0 | 25 | 0.8 |
| IPO9 | 9 | 1 | 0 | 0 | 0 | 10 | 0.4 |
| IPO11 | 18 | 2 | 0 | 0 | 0 | 20 | 0.6 |
| IPO13 | 14 | 1 | 0 | 0 | 0 | 15 | 0.5 |
| TNPO1 | 12 | 6 | 0 | 0 | 0 | 18 | 0.6 |
| TNPO2 | 10 | 1 | 0 | 0 | 0 | 11 | 0.4 |
| TNPO3 | 15 | 3 | 0 | 0 | 0 | 18 | 0.6 |
| XPOT | 28 | 1 | 3 | 0 | 0 | 32 | 1 |
| XPO4 | 17 | 3 | 0 | 3 | 0 | 23 | 0.9 |
| XPO5 | 17 | 4 | 0 | 0 | 0 | 21 | 0.7 |
| XPO6 | 14 | 2 | 0 | 0 | 0 | 16 | 0.6 |
| XPO7 | 8 | 2 | 0 | 2 | 0 | 12 | 0.4 |

Supplemental Table 6: q-value of the correlation between nuclear transport receptor genes and cell cycle genes in PCAWG pan-cancer dataset.

|  | CDK1 | CDK2 | CDK4 | CDK6 | CCNA1 | CCNB1 | CCND1 | CCNE2 |
| --- | --- | --- | --- | --- | --- | --- | --- | --- |
| KPNA1 | 2.86E-07 | 4.74E-09 | 0.397 | 6.82E-07 | 0.146 | 0.0517 | 0.0125 | 4.32E-41 |
| KPNA2 | 7.52E-266 | 5.05E-140 | 5.05E-140 | 2.24E-07 | 6.55E-09 | 2.00E-278 | 3.39E-14 | 1.30E-116 |
| KPNA3 | 2.54E-27 | 2.34E-10 | 1.35E-09 | 2.95E-42 | 0.836 | 5.54E-21 | 0.0111 | 7.14E-46 |
| KPNA4 | 9.20E-41 | 1.21E-33 | 3.50E-19 | 1.26E-06 | 2.18E-27 | 3.08E-27 | 0.482 | 5.52E-29 |
| KPNA5 | 0.0364 | 0.798 | 1.47E-06 | 0.517 | 0.0206 | 4.82E-08 | 3.00E-07 | 0.0206 |
| KPNA6 | 0.0778 | 7.66E-07 | 0.0647 | 0.589 | 9.09E-11 | 9.09E-11 | 1.07E-14 | 3.80E-05 |
| KPNA7 | 3.14E-05 | 0.89 | 0.285 | 0.279 |  |  | 0.353 | 6.12E-04 |
| KPNB1 | 3.86E-66 | 1.81E-70 | 1.43E-26 | 5.48E-23 | 0.176 | 2.82E-54 | 1.37E-07 | 8.55E-73 |
| IPO4 | 2.35E-22 | 1.67E-18 | 2.87E-11 | 1.72E-03 | 2.20E-07 | 2.15E-39 | 0.866 | 0.644 |
| IPO5 | 9.64E-27 | 1.79E-22 | 6.02E-30 | 2.95E-19 | 0.122 | 1.55E-30 | 0.0955 | 3.63E-27 |
| IPO7 | 3.73E-11 | 3.59E-05 | 7.98E-08 | 4.25E-15 | 0.514 | 5.41E-09 | 0.0273 | 7.97E-29 |
| IPO8 | 0.811 | 5.74E-03 | 0.828 | 1.34E-10 | 1.82E-01 | 0.0313 | 0.162 | 5.78E-05 |
| TNPO1 | 2.57E-10 | 4.82E-07 | 0.0877 | 1.46E-33 | 0.14 | 0.0126 | 0.0589 | 9.57E-41 |
| TNPO2 | 0.154 | 2.90E-06 | 1.53E-11 | 0.0138 | 4.15E-12 | 0.0227 | 0.861 | 0.131 |
| XPOT | 5.04E-48 | 1.28E-35 | 6.30E-48 | 2.36E-14 | 1.10E-09 | 8.31E-30 | 9.58E-03 | 4.75E-44 |
| XPO4 | 2.32E-13 | 6.25E-12 | 0.578 | 2.26E-32 | 2.16E-03 | 8.38E-07 | 3.70E-03 | 4.76E-40 |
| XPO5 | 1.35E-47 | 6.60E-46 | 3.44E-43 | 1.59E-11 | 5.88E-12 | 3.92E-56 | 0.555 | 7.30E-34 |
| XPO6 | 1.34E-19 | 1.21E-21 | 5.67E-23 | 1.52E-08 | 5.87E-04 | 4.17E-27 | 1.56E-08 | 1.12E-14 |
| XPO7 | 1.19E-04 | 1.32E-11 | 1.30E-10 | 2.59E-03 | 1.46E-13 | 2.55E-05 | 0.658 | 5.94E-09 |

Supplemental Table 7: q-value of the correlation between nuclear transport receptor genes and cell cycle genes in CCLE dataset.

|  | CDK1 | CDK2 | CDK4 | CDK6 | CCNA1 | CCNB1 | CCND1 | CCNE2 |
| --- | --- | --- | --- | --- | --- | --- | --- | --- |
| KPNA2 | 1.87E-39 | 2.99E-34 | 1.79E-19 | 1.86E-02 | 1.47E-04 | 4.36E-74 | 3.04E-03 | 8.46E-22 |
| KPNA3 | 1.62E-19 | 2.84E-12 | 2.40E-10 | 1.21E-02 | 7.30E-01 | 5.99E-15 | 4.92E-01 | 2.23E-12 |
| KPNA4 | 1.17E-03 | 7.36E-06 | 0.625 | 0.43 | 0.191 | 3.49E-12 | 3.23E-09 | 0.574 |
| KPNA5 | 1.33E-09 | 1.82E-06 | 0.619 | 0.238 | 8.98E-03 | 1.96E-05 | 3.94E-14 | 1.38-15 |
| KPNA6 | 2.53E-03 | 6.11E-08 | 0.45 | 0.0109 | 3.00E-06 | 2.19E-09 | 4.54E-01 | 3.39E-07 |
| KPNA7 | 4.23E-17 | 1.44E-17 | 4.08E-14 | 4.90E-05 | 6.28E-07 | 5.89E-02 | 2.24E-19 | 6.24E-01 |
| KPNB1 | 3.84E-17 | 2.84E-33 | 4.65E-36 | 5.11E-01 | 8.05E-02 | 1.69E-26 | 1.52E-01 | 4.04E-28 |
| IPO5 | 2.78E-17 | 3.13E-15 | 6.54E-20 | 1.23E-01 | 5.54E-01 | 1.13E-07 | 1.04E-04 | 5.91E-15 |
| IPO7 | 2.23E-12 | 7.67E-04 | 1.24E-09 | 5.11E-01 | 1.91E-01 | 9.41E-08 | 5.01E-03 | 9.88E-05 |
| IPO8 | 6.56E-01 | 6.37E-16 | 6.48E-07 | 7.99E-01 | 4.59E-01 | 4.95E-06 | 2.26E-01 | 3.43E-17 |
| IPO9 | 1.94E-19 | 6.87E-28 | 1.04E-15 | 1.13E-02 | 8.44E-05 | 2.71E-07 | 3.39E-01 | 1.29E-42 |
| IPO11 | 7.16E-29 | 7.54E-14 | 7.59E-23 | 1.27E-01 | 6.80E-01 | 5.20E-63 | 8.98E-01 | 3.04E-13 |
| IPO13 | 3.78E-02 | 1.73E-05 | 6.87E-03 | 1.54E-01 | 1.72E-07 | 9.93E-01 | 2.70E-05 | 9.43E-10 |
| TNPO1 | 1.14E-09 | 2.83E-05 | 1.29E-13 | 4.91E-01 | 4.18E-02 | 3.70E-32 | 8.93E-03 | 7.64E-10 |
| TNPO2 | 2.28E-03 | 5.78E-18 | 4.11E-14 | 9.74E-01 | 1.78E-03 | 1.07E-01 | 2.96E-07 | 1.17E-20 |
| TNPO3 | 1.06E-01 | 9.48E-12 | 3.97E-10 | 1.09E-04 | 9.90E-01 | 7.80E-01 | 1.46E-05 | 1.01E-05 |
| XPOT | 2.91E-06 | 1.10E-04 | 3.96E-16 | 8.34E-01 | 1.61E-01 | 9.41E-03 | 2.34E-07 | 4.27E-01 |
| XPO4 | 2.85E-11 | 1.84E-16 | 8.25E-12 | 3.43E-02 | 8.69E-03 | 9.47E-01 | 1.87E-06 | 2.12E-16 |
| XPO5 | 3.58E-08 | 7.41E-16 | 6.49E-17 | 2.86E-01 | 1.08E-01 | 3.12E-01 | 1.23E-11 | 2.60E-16 |
| XPO6 | 1.78E-02 | 1.28E-14 | 1.81E-12 | 5.49E-02 | 4.99E-01 | 1.35E-02 | 4.50E-08 | 3.70E-08 |
| XPO7 | 2.13E-08 | 5.54E-19 | 4.08E-19 | 3.73E-01 | 3.22E-02 | 2.46E-01 | 1.07E-11 | 2.12-18 |

Supplemental Table 8: Mutation types of each nuclear transport receptor in CCLE dataset

|  | Missense | Truncating | Inflame | Splice | Fusion | Total | % |
| --- | --- | --- | --- | --- | --- | --- | --- |
| KPNA1 | 3 | 2 | 0 | 0 | 0 | 5 | 0.8 |
| KPNA2 | 6 | 1 | 0 | 0 | 0 | 7 | 1 |
| KPNA3 | 3 | 0 | 0 | 1 | 0 | 4 | 0.5 |
| KPNA4 | 3 | 0 | 0 | 1 | 1 | 5 | 0.8 |
| KPNA5 | 2 | 0 | 0 | 0 | 0 | 2 | 0.3 |
| KPNA6 | 5 | 0 | 0 | 0 | 1 | 6 | 1 |
| KPNA7 | 0 | 0 | 0 | 0 | 0 | 0 | - |
| IPO4 | 1 | 0 | 1 | 0 | 0 | 1 | 0.2 |
| IPO5 | 3 | 0 | 0 | 0 | 0 | 3 | 0.5 |
| IPO7 | 9 | 2 | 0 | 2 | 0 | 13 | 1.8 |
| IPO8 | 4 | 4 | 0 | 3 | 0 | 11 | 1.6 |
| IPO9 | 4 | 0 | 0 | 0 | 1 | 5 | 0.8 |
| IPO11 | 5 | 1 | 0 | 0 | 0 | 6 | 1 |
| IPO13 | 6 | 2 | 0 | 0 | 0 | 8 | 1.3 |
| TNPO1 | 3 | 0 | 0 | 0 | 0 | 3 | 0.6 |
| TNPO2 | 6 | 0 | 0 | 0 | 0 | 6 | 1.2 |
| TNPO3 | 2 | 1 | 0 | 0 | 0 | 3 | 0.6 |
| XPOT | 5 | 2 | 0 | 0 | 0 | 7 | 1.4 |
| XPO4 | 5 | 1 | 0 | 0 | 0 | 6 | 1.2 |
| XPO5 | 4 | 0 | 0 | 0 | 0 | 4 | 0.8 |
| XPO6 | 4 | 0 | 0 | 0 | 0 | 4 | 0.8 |
| XPO7 | 5 | 1 | 0 | 0 | 0 | 6 | 1.2 |
